# Editorial for Special Issue “Lactic Acid Bacteria, Biopreservation Agents for Fruit and Vegetables”

**DOI:** 10.3390/microorganisms9050939

**Published:** 2021-04-27

**Authors:** Fabienne Remize, Charlène Leneveu-Jenvrin, Cyrielle Garcia

**Affiliations:** QualiSud, Université La Réunion, CIRAD, Université Montpellier Institut Agro Montpellier, Université Avignon, F-97490 Saint Pierre, France; charlene.leneveu@univ-reunion.fr (C.L.-J.); cyrielle.garcia@univ-reunion.fr (C.G.)

Amongst the microbial diversity in the food chain, lactic acid bacteria (LAB) are in the front row for their positive roles. They contribute to enhance the shelf life of many different food products through their fermentative activity or their ability to limit the development of other microorganisms. Their panel of activity is involved in the modification of food properties and they are also recognized for their probiotic activities.

Many fruits and vegetables are traditionally lactic acid fermented. However, the current research on LAB opens new perspectives, related to the detailed examination of how LAB modify technological, nutritional or sensory properties of foods. The Special Issue “Lactic Acid Bacteria, Biopreservation Agents for Fruit and Vegetables” contains 11 research articles and one review, which perfectly illustrate the new paradigms on the use of LAB to preserve fruit and vegetable quality. 

In this Issue, lactic acid fermentation was applied to different food products within the framework of various objectives, but all related to better health expectations. Fruits and vegetables are substrates of interest for the diet as a high consumption over time significantly contributes to limit chronic diseases. A large set of drinks based on tomato and including different fruit or plant substrates was designed and the drinks were assessed according to their antioxidant activity [1]. In a similar approach, lactic acid fermentation of African nightshade leaves not only increased the shelf life but also the antioxidant activity and the bioavailability of flavonoids [2]. Schettino et al. [3] showed that addition of fermented chickpea flour to fresh pasta provided better nutritional properties. Moreover, chickpea sourdough was efficient in limiting the development of yeasts and molds and, to a lesser extent, of enterobacteria, hence increasing the shelf life. Lastly, a study showed that lactic-fermented or non-fermented papaya pulp inhibited human cell infection by Zika virus and reduced the virus progeny production in infected cells [4].

Probiotic, often LAB, survival is another issue to be addressed to provide functional foods. Dried apple slices were processed with a vacuum impregnation step to add trehalose as a microbial protectant and probiotics, and survival through the drying step and storage was compared [5]. The impact of the drying process appeared of crucial importance for probiotic survival during storage, and LAB encapsulation provides an additional protective effect for intestinal tract survival of probiotics [6]. Related to the concern of probiotic survival, the use of industrial by-products from tropical fruit processing was shown to be an effective way to maintain LAB viability during freeze drying and storage [7].

In food production, lactic acid fermentation can be seen as a processing step. However, in many food products, the typical fermentative flavors and acidification are not wanted. This is the case of minimally processed fruits and vegetables which should retain, during their marketing, the aspect and taste of fresh materials. LAB can contribute, through the production of bacteriocins, short 10–30-amino acid residue peptides. If the addition of bioprotective cultures to minimally processed foods is still challenging due to the many scientific bottlenecks, the design of active packaging containing bacteriocins is promising [8]. Acidification is a sign of sensory quality loss, which can result from temperature abuse. To limit this unwanted loss of sensory quality, addition of attenuated LAB is another approach to both provide probiotics and preserve the sensory quality of non-dairy vegetable beverages [9].

Food processing can involve high-hydrostatic pressure treatments to inactivate foodborne pathogens or spoilage microorganisms. However, this non-thermal pasteurization method can modify the protein profile of bacteria, leading then to misidentification with the rapid MALDI-TOF MS (matrix-assisted laser desorption ionization time-of-flight mass spectrometry) method [10].

Animal feed including ruminant silage is obtained by anaerobic bioprocessing of grass forage. LAB can be added for the fermentation step. Araújo et al. [11] selected LAB from silage based on their ability to produce a carboxyesterase, an enzyme involved in hydrolysis of fibers, thus improving silage digestibility, or to inhibit the development of the mycotoxin producer *Fusarium graminearum.* In another study [12], *Lactobacillus* supplementation to silage was less efficient than other additives, such as pectin or pectinase, which modulate the rumen microbial diversity towards a higher relative abundance of *Leuconostoc*, and *Bacillus* and *Aeromonas*.

The Special Issue gathers together papers illustrating the diversity of approaches involving LAB to increase the quality of fruit or vegetable food and feed products.

## Data Availability

Not applicable.
